# Clinical impact of healthcare-associated respiratory syncytial virus in hospitalized adults

**DOI:** 10.1017/ice.2022.128

**Published:** 2023-03

**Authors:** Alexandra Hill-Ricciuti, Edward E. Walsh, William G. Greendyke, Yoonyoung Choi, Angela Barrett, Luis Alba, Angela R. Branche, Ann R. Falsey, Matthew Phillips, Lyn Finelli, Lisa Saiman

**Affiliations:** 1 Department of Pediatrics, Columbia University Irving Medical Center, New York, New York; 2 Department of Medicine, University of Rochester, Rochester, New York; 3 Rochester General Hospital, Rochester, New York; 4 Department of Medicine, Columbia University Irving Medical Center, New York, New York; 5 Department of Infection Prevention & Control, New York-Presbyterian Hospital, New York, New York; 6 Center for Observational and Real-World Evidence, Merck & Company, Kenilworth, New Jersey

## Abstract

**Objective:**

To describe the clinical impact of healthcare-associated (HA) respiratory syncytial virus (RSV) in hospitalized adults.

**Design:**

Retrospective cohort study within a prospective, population-based, surveillance study of RSV-infected hospitalized adults during 3 respiratory seasons: October 2017–April 2018, October 2018–April 2019, and October 2019–March 2020.

**Setting:**

The study was conducted in 2 academically affiliated medical centers.

**Patients:**

Each HA-RSV patient (in whom RSV was detected by PCR test ≥4 days after hospital admission) was matched (age, sex, season) with 2 community-onset (CO) RSV patients (in whom RSV was detected ≤3 days of admission).

**Methods:**

Risk factors and outcomes were compared among HA-RSV versus CO-RSV patients using conditional logistic regression. Escalation of respiratory support associated with RSV detection (day 0) from day −2 to day +4 was explored among HA-RSV patients.

**Results:**

In total, 84 HA-RSV patients were matched to 160 CO-RSV patients. In HA-RSV patients, chronic kidney disease was more common, while chronic respiratory conditions and obesity were less common. HA-RSV patients were not more likely to be admitted to an ICU or require mechanical ventilation, but they more often required a higher level of care at discharge compared with CO-RSV patients (44% vs 14%, respectively). Also, 29% of evaluable HA-RSV patients required respiratory support escalation; these patients were older and more likely to have respiratory comorbidities, to have been admitted to intensive care, and to die during hospitalization.

**Conclusions:**

HA-RSV in adults may be associated with escalation in respiratory support and an increased level of support in living situation at discharge. Infection prevention and control strategies and RSV vaccination of high-risk adults could mitigate the risk of HA-RSV.

The impact of respiratory syncytial virus (RSV) in adults is increasingly appreciated. RSV can cause significant morbidity in older adults and in those who are immunocompromised or have cardiopulmonary comorbidities.^1,2^ The healthcare costs of RSV-related hospitalizations in adults are similar to those of influenza.^3^ Although healthcare-associated (HA) influenza in hospitalized adults has been well described and is known to result in adverse clinical outcomes and increased healthcare utilization,^4–6^ less is known about HA-RSV. Although several studies have described RSV outbreaks in a variety of healthcare settings caring for adults,^7–14^ risk factors and outcomes associated with HA-RSV in nonoutbreak settings have not been well characterized.

To address this knowledge gap, we assessed the demographic characteristics, comorbid conditions, and clinical outcomes of adult patients with HA-RSV compared with adult patients hospitalized with community-onset (CO) RSV infections. As a potential surrogate for severity of illness in those with HA-RSV, we explored escalation of respiratory support associated with detection of RSV, and we compared the characteristics and outcomes of patients with HA-RSV who did and did not have escalation of respiratory support.

## Methods

### Study design, sites, and participants

We designed a retrospective cohort study within a large, prospective, multicenter, multiseason, population-based, active surveillance study of RSV-associated hospitalization in adults 18 years of age and older.^15^ As previously described, active surveillance for RSV took place during 3 successive RSV seasons from October 2017 to March 2020. To identify patients with laboratory-confirmed CO-RSV infection, study staff reviewed infection control databases and clinical virology laboratory logs to ascertain the results of PCR tests ordered as the standard of care for patients admitted with acute respiratory infection. To identify missed patients with CO-RSV and patients with HA-RSV infection, when the surveillance seasons ended, the electronic medical record (EMR) was queried for all positive RSV tests in hospitalized adults during the study period. The study sites were academically affiliated hospital systems in northern Manhattan and Rochester, New York. In the current retrospective study, we compared risk factors and outcomes for adult patients with HA-RSV with hospitalized adults with CO-RSV. The institutional review boards of the study sites approved this study with a waiver of informed consent.

### HA-RSV and CO-RSV case definitions and matching criteria

HA-RSV was defined as a patient hospitalized for 4 or more calendar days at a study site prior to RSV detection or transferred from an acute-care hospital with a combined length of hospital stay at either the outside hospital or study site of 4 or more contiguous days prior to RSV detection. Patients transferred from outside hospitals with known RSV infection were excluded. Patients with CO-RSV were selected from the previously described cohort of adults hospitalized with CO-RSV.^15^ Eligible patients with CO-RSV were ≥18 years of age, had symptoms consistent with acute respiratory illness, and had RSV detected within 3 calendar days of admission. Each patient with HA-RSV was matched to 2 patients with CO-RSV by age (±5 years), sex, and RSV season. If >2 suitable CO-RSV patients were identified for an HA-RSV patient, those closest in age to the HA-RSV patient were selected.

### Viral detection

The northern Manhattan study sites used the FilmArray Respiratory Panel (BioFire Diagnostics, Salt Lake City, UT), which detects influenza types A H3, A H1, B; parainfluenza virus types 1–4; RSV; human metapneumovirus; adenovirus, rhinovirus and enterovirus; and human coronavirus types 229E, HKU1, NL63, OC43; as well as *Mycoplasma pneumoniae*, *Bordatella pertussis*, and *Chlamydophilia pneumoniae*. The Rochester study sites used either the FilmArray Respiratory Panel, Simplexa FLU/RSV Duplex (Diasorin Molecular, Cypress, CA) or Cepheid GeneXpert Flu/RSV Duplex (Cepheid, Sunnyvale, CA).

### Data collection and outcomes

For HA-RSV patients, the reasons for hospitalization and for respiratory pathogen testing, such as worsening cough, were extracted from healthcare providers’ progress notes. *International Classification of Disease, Tenth Revision* (ICD-10) discharge codes that were related to RSV (ie, B97.4, J12.1, J20.5, or J21.0) were extracted from the EMR, and cause(s) of death were abstracted from the death certificate or death notes, when applicable.

For both HA-RSV and CO-RSV patients, demographic, and clinical characteristics, including comorbid conditions and living situation at admission, were collected. Living situation was classified as living independently at home, living at home with assistance from family members or home health aide or residing in an assisted living facility, or living in a rehabilitation or skilled nursing facility.^16^ Patients transferred from acute-care hospitals or who were homeless were excluded from assessment of living situation at admission.

For both HA-RSV and CO-RSV patients, outcomes included length of stay after RSV detection, admission to an intensive care unit (ICU) and/or mechanical ventilation initiated in the 4 days following RSV detection, and in-hospital mortality. For those who survived, living situation at discharge and changes in living situation from admission to discharge that reflected the need for increased support (eg, living independently at admission versus discharge to a skilled nursing facility) were determined as previously described.^16^ Patients who died, were transferred to another acute-care hospital, or were not eligible for the analysis of living situation at admission were excluded from the analysis of changes in living situation. Readmission within 30 days of discharge was also assessed.

### Escalation of respiratory support

To explore escalation of respiratory support associated with HA-RSV, the type of respiratory support modalities (eg, nasal cannula and/or mechanical ventilation) and degree of support (ie, fraction of inspired oxygen [FiO_2_] and mean airway pressure [MAP] sustained for ≥1 hour each day) were collected before and after detection of RSV (day of detection = day 0). The daily maximum respiratory support used from day −10 (when available) to day −3 prior to RSV detection was considered the baseline support. This support was compared with the daily maximum respiratory support used from day −2 to day +4. This timeframe was selected because it reflected the potential time course of clinical deterioration from RSV prior to and after providers sent the diagnostic test. Patients who were transferred to the study sites from day −2 to day 0 were excluded from this analysis because a baseline period of support could not be reliably established.

Escalation of respiratory support was defined as follows: (1) increase in supplemental oxygen by ≥ 1 liter per minute for ≥ 1 hour while maintaining the same mode of noninvasive respiratory support, (2) increase in modality of noninvasive support such as transition from room air to nasal cannula and/or nasal cannula to bilevel positive airway pressure (BiPAP), (3) transition from noninvasive to invasive support such as BiPAP to mechanical ventilation, or (4) increase in invasive support such as increase in MAP and/or FiO_2_ while mechanically ventilated.

### Statistical analysis

Baseline demographic and clinical characteristics and outcomes of patients with HA-RSV versus CO-RSV were compared using conditional logistic regression. Outcomes with *P* < .10 in univariate analysis were then assessed in a multivariable logistic regression model after controlling for comorbid conditions that were significantly different between patients with HA-RSV and CO-RSV. Odds ratios and 95% confidence intervals were also calculated. Length of stay following RSV detection in patients with HA-RSV was compared with patients with CO-RSV but was not included in the bivariate or multivariable analysis due to confounding by comorbid conditions, particularly in patients with HA-RSV.

The proportion of patients with HA-RSV with and without escalation of respiratory support was determined, and the types and timing of escalations were characterized. Clinical and demographic characteristics of patients with HA-RSV with and without escalation of respiratory support were compared using the χ^2^ test or the Fisher exact test, as appropriate, for categorical variables and the Mann-Whitney *U* test or the Student *t* test as appropriate for continuous variables. Outcomes with *P* < .10 in univariate analysis were then assessed in a multivariable logistic regression model. All analyses were conducted in SAS version 9.4 software (SAS Institute, Cary, NC) and *P* < .05 was considered statistically significant.

## Results

### Characteristics of patients with HA-RSV versus CO-RSV

**Fig. 1.**
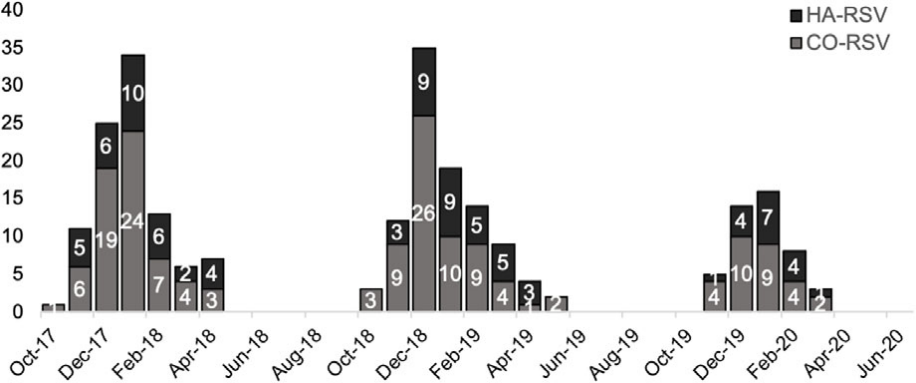
Epidemiology of 84 HA-RSV versus 160 CO-RSV cases in 3 respiratory viral seasons: October 2017–April 2018, October 2018–April 2019, and October 2019–March 2020. Due to the coronavirus disease 2019 (COVID-19) pandemic, data collection in the third season ceased in March 2020 due to the onset of the COVID-19 pandemic and cessation of testing for non–severe acute respiratory coronavirus virus 2 (SARS-CoV-2) viruses at the study sites. Note. HA, healthcare-associated; RSV respiratory syncytial virus; CO, community onset.

During the study period, 84 patients met the HA-RSV case definition including 33 patients between October 2017 and April 2018, 34 patients between October 2018 and April 2019, and 17 patients between October 2019 and March 2020 (Fig. 1). Patients with HA-RSV were admitted to the study sites for management of a variety of conditions, most commonly cardiac conditions (23%), neurologic or psychiatric events (19%), and infections (14%). Also, 16 (19%) were transferred from another acute-care hospital. Patients with HA-RSV were hospitalized for a median of 12 days (IQR, 7–16 days) prior to detection of RSV. They underwent testing for respiratory pathogens at the study sites due to new onset or worsening respiratory symptoms or physical findings, most commonly cough (51%), fever (30%), shortness of breath (13%), and/or rhonchi (13%). No RSV outbreaks or clusters were identified by the infection prevention and control teams at the sites during the study period.

Of the 84 patients with HA-RSV, 76 were matched to 2 patients with CO-RSV and 8 were matched to 1 patient because an appropriate second CO-RSV match could not be found, for a total of 160 patients with CO-RSV. The demographic characteristics were similar between the 2 groups (Table 1). Most patients with HA-RSV (86%) and CO-RSV (87%) had ≥1 comorbid condition, most commonly cardiac comorbidities in both groups. Patients with HA-RSV were more likely to have chronic kidney disease (CKD, 31% vs 26%; *P* = .04) and were less likely to have respiratory comorbidities (31% vs 46%; *P* = .03) and/or obesity (23% vs 32%; *P* = .04).

**Table 1.** Characteristics of Patients With Healthcare-Associated (HA) Respiratory Syncytial Virus (RSV) Versus Community-Onset (CO) RSV, Univariate Analysis

Characteristic	HA-RSV (n=84), No. (%)	CO-RSV (n=160), No. (%)	*P* Value
**Demographic characteristics**			
Sex, male	41 (51)	79 (50)	1.00
**Age group**			1.00
18–49 y (ref)	14 (17)	23 (14)	…
50–64 y	29 (35)	54 (34)	1.00
≥65 y	41 (49)	83 (52)	1.00
**Race**			
White (ref)	38 (46)	67 (42)	…
Black/African American	17 (20)	28 (18)	.87
Asian	1 (1)	0 (0)	.99
Unknown/Other	28 (33)	65 (41)	.46
**Ethnicity**			
Hispanic (ref)	18 (21)	50 (31)	…
Non-Hispanic	41 (49)	47 (29)	.35
Unknown	25 (30)	63 (39)	**.03**
**Living situation at admission** ^a^			
Living independently (ref)	38 (58)	89 (59)	…
Living at home with assistance of family/friends or aide or in assisted living facility	19 (29)	52 (34)	.43
Skilled nursing or rehabilitation facility	9 (14)	11 (7)	.06
**Clinical characteristics, no. (%)**			
**Comorbid conditions**			
Respiratory^b^	26 (31)	74 (46)	**.03**
Cardiac^c^	51 (61)	77 (48)	.75
Chronic kidney disease	26 (31)	41 (26)	**.04**
Immunosuppressive conditions^d^	28 (33)	54 (34)	.81
Neurologic	22 (26)	28 (18)	.92
Chronic liver disease	6 (7)	5 (3)	.11
Diabetes	22 (26)	56 (35)	.21
Obesity	19 (23)	51 (32)	**.04**
**No. of comorbid conditions**			
0 (ref)	12 (14)	20 (13)	…
1–2	41 (49)	85 (53)	.24
≥3	31 (37)	55 (34)	.17

Note. Bold *P* value indicates statistical significance.

a Living status at admission excludes patients who were transferred in from other acute-care hospitals (n=17), those who were homeless (n=3), and those missing living status at admission (n=6).

b Respiratory conditions included chronic obstructive pulmonary disease, asthma, pulmonary hypertension, and obstructive sleep apnea.

c Cardiac conditions included congestive heart failure, coronary artery disease, hypertension, arrythmias, and valvular heart disease.

d Immunosuppressive conditions included HIV, cancer, and transplant recipient.

### Outcomes of patients with HA-RSV versus CO-RSV

Following RSV detection, the median length of hospitalization for patients with HA-RSV was longer than that for patients with CO-RSV (10 days [IQR, 5–21] for HA-RSV vs 6 days [IQR, 3–10] for CO-RSV; *P* < .001). Although not statistically significant, the proportion of patients who died during hospitalization was higher among those with HA-RSV than those with CO-RSV (15% vs 6%; *P* = .25) (Table 2). Among those who survived to discharge and had an admission living situation available, patients with HA-RSV were more likely to require an increased level of support in their living situation at discharge compared with their living situation at admission than patients with CO-RSV (42% vs 14%; *P* = .01). In multivariable analysis, after controlling for comorbid conditions, patients with HA-RSV remained more likely to require an increased level of support at discharge compared with patients with CO-RSV (OR, 6.96; 95% CI, 1.39–34.78; *P* = .02).

**Table 2.** Outcomes of Patients With Healthcare-Associated (HA) Respiratory Syncytial Virus (RSV) Versus Community-Onset (CO) RSV, Univariate Analysis

Outcomes	HA-RSV (n=84), No. (%)	CO-RSV (n=160), No. (%)	*P* Value
Admission to ICU within 4 d following RSV detection	13 (15)	31 (20)	.60
Ventilation initiated within 4 d following RSV detection	3 (4)^a^	16 (18)	.52
In-hospital mortality	13 (15)	9 (6)	.25
**Living situation at discharge** ^**b**^			
Living independently (ref)	18 (26)	72 (50)	…
Living at home with assistance of family/friends or aide or in an assisted living facility	20 (28)	48 (34)	.24
Skilled nursing or rehabilitation facility	30 (44)	23 (16)	**<.001**
**Changes in living situation from admission to discharge** ^**c**^,^**d**^			
Unchanged (ref)	31 (56)	122 (86)	…
Increased level of support	24 (44)	20 (14)	**.01**
Readmission within 30 d	14 (17)	15 (9)	.99

Note. ICU, intensive care unit. Bold *P* value indicates statistical significance.

a One patient excluded from analysis of escalation of respiratory support.

b Excludes patients who died (n=22), were homeless at discharge (n=3), were transferred to another acute care hospital (n=2), and those for whom data were unavailable (n=6).

c Excludes patients who died (n=22), were transferred to study sites but survived to discharge (n=13), those who were homeless at admission and/or discharge (n=3), were transferred to another acute care hospital (n=2), and those for whom living status at admission and/or discharge were unavailable (n=7).

d No patients had a decreased level of support at discharge.

### Escalation of respiratory support among patients with HA-RSV

**Fig. 2.**
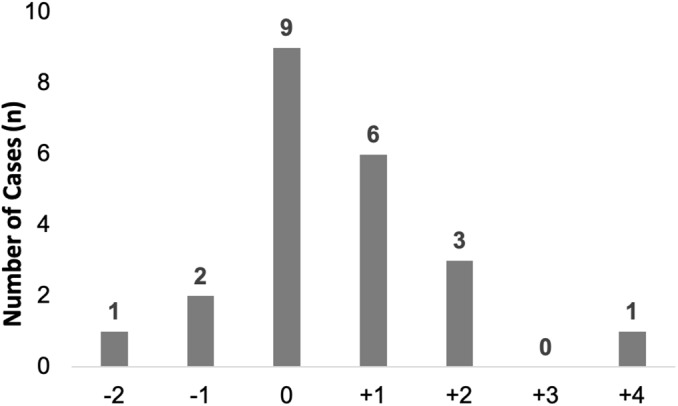
Timing of respiratory support escalation relative to HA-RSV detection date. During the interval day +2 to day −4, the number of HA-RSV cases with escalation of respiratory support (first day of escalation) is shown. Day 0 is the day of detection of RSV. Note. HA, healthcare-associated; RSV respiratory syncytial virus.

Overall, 77 evaluable patients (92%) with HA-RSV were included in the analysis of escalation of respiratory support (Table 3). From day −2 to day +4 relative to RSV detection, 55 (71%) did not have an escalation in respiratory support, including 44 who remained on room air. Of the 22 (29%) who had escalation of respiratory support from their baseline, 11 (50%) were changed from room air to nasal cannula, 4 (18%) had an increase in FiO_2_ on nasal cannula, 2 (9%) were changed from nasal cannula to a non-rebreather mask, 1 (5%) was changed from room air to BiPAP, 2 (9%) had mechanical ventilation initiated (1 from room air and 1 from nasal cannula), and 2 (9%) had an increase in MAP and/or FiO_2_ on mechanical ventilation. Of 22 escalations, 15 (68%) occurred on day 0 or day +1 (Fig. 2).

**Table 3.** Comparison of Healthcare-Associated (HA) Respiratory Syncytial Virus (RSV) Cases With and Without Respiratory Support Escalation, Univariate Analysis

Characteristic	Escalation (n=22), No. (%)	No Escalation (n=55), No. (%)	*P* Value
Sex, male	12 (55)	26 (50)	.72
Age, median y (IQR)	73 (65–78)	62 (51–70)	**<.001**
Age group			
18–49 y	1 (5)	12 (22)	…
50–64 y	5 (23)	23 (42)	.65
≥65 y	16 (73)	20 (36)	**.02**
**Race**			
White (ref)	11 (50)	24 (44)	…
Black/African American	5 (23)	12 (22)	1.00
Asian	0 (0)	0 (0)	1.00
Unknown/Other	6 (27)	18 (34)	.77
**Ethnicity**			
Hispanic (ref)	4 (18)	13 (24)	…
Non-Hispanic	14 (63)	26 (47)	.54
Unknown	4 (18)	16 (29)	1.00
**Living situation at admission** ^**a**^			
Living independently (ref)	9 (53)	29 (62)	…
Living at home with assistance of family/friends or aide	4 (24)	13 (28)	1.00
Skilled nursing or rehabilitation facility	4 (24)	5 (11)	.24
**Comorbid conditions**			
Respiratory	11 (50)	10 (18)	**.005**
Cardiac	12 (55)	34 (62)	.61
Chronic kidney disease	9 (41)	14 (25)	.18
Immunosuppressive conditions	9 (41)	19 (35)	.61
Neurologic	6 (27)	15 (27)	1.00
Chronic liver disease	1 (5)	4 (7)	1.00
Diabetes	6 (27)	16 (29)	.87
Obesity	5 (23)	12 (22)	1.00
**No. of comorbid conditions**			
0 (ref)	2 (9)	9 (16)	…
1–2	11 (50)	28 (51)	.70
≥3	9 (41)	18 (33)	.45
**Outcomes**			
ICU admission within 4 days following RSV detection	8 (36)	2 (4)	**<.001**
Median total hospital length of stay, d (IQR)	29 (24–32)	20 (13–50)	.47
Median length of stay following RSV detection, d (IQR)	15 (8–22)	8 (4–21)	**.03**
In-hospital mortality	6 (27)	4 (7)	**.03**
Readmission within 30 d	5 (23)	8 (15)	.50
**Living situation at discharge** ^**b**^			
Living independently (ref)	5 (18%)	13 (27%)	…
Living at home with assistance of family/friends or aide	4 (24)	16 (33)	1.00
Skilled nursing or rehabilitation facility	9 (59)	19 (40)	.49

Note. IQR, interquartile range, ICU, intensive care unit. Bold *P* value indicates statistical significance.

a Living situation on admission excludes patients who were transferred to study sites (n=11), homeless (n=1), and those for whom data were unavailable (n=1).

b Living situation at discharge excludes patients who died during admission (n=10), those transferred to another other acute care hospital (n=1), those homeless (n=1), and those for whom data were unavailable (n=1).

### HA-RSV with and without escalation of respiratory support

Patients with HA-RSV who had an escalation of respiratory support were significantly older than those without escalation (median age, 73 years [IQR, 65–78] vs 62 years [IQR, 51–70]; *P* < .001) and were more likely to have respiratory comorbidities (50% vs 18%; *P* = .005) (Table 3). Patients with HA-RSV with escalations were more likely to have been admitted to an ICU (27% vs 7%; *P* < .001), to have had a longer length of stay following RSV detection (median, 15 days [IQR, 8–22] vs 8 days [IQR, 4–21]; *P* = .03), and to have died during hospitalization (36% vs 4%; *P* = .03). In multivariable analysis, after controlling for age and respiratory conditions, ICU admission (*P* = .03) and mortality (*P* = .002) remained associated with escalation of respiratory support, but length of stay after RSV detection was no longer significantly different in those with and without escalation (*P* = .48).

### Causes of death and analysis of discharge codes for HA-RSV

Of the 79 (94%) of 84 patients with HA-RSV with ICD-10 discharge codes available, 14 (18%) had an RSV-related diagnostic code. Only 1 of the 17 patients with an escalation of respiratory support had ICD-10 discharge codes available. Of the 13 patients with HA-RSV who died during admission, none had RSV listed as a cause or contributor to mortality on their death certificates.

## Discussion

In this study, we assessed factors associated with HA-RSV in hospitalized adults in nonoutbreak settings, and we noted some interesting differences in patterns of comorbid conditions when we compared our study to similar studies conducted in HA versus CO influenza. Patients with HA influenza, compared with patients with CO influenza, had more chronic medical conditions, including higher rates of chronic lung disease other than asthma, cardiovascular diseases, metabolic disease, and immunosuppressive conditions.^17^ In contrast, patients with HA-RSV were less likely than patients with CO-RSV to have respiratory comorbidities.

Respiratory comorbidities are well known to be exacerbated by RSV infection, often leading to subsequent hospitalization.^18–21^ In the large prospective surveillance study from which the matched patients with CO-RSV were derived, we found that persons with chronic obstructive pulmonary disease and asthma had higher hospitalization rates due to CO-RSV infection than those without these conditions.^15^ In the current study, we noted that those with CO-RSV were more likely to be obese; however, previous studies have not identified obesity as a risk factor for severe RSV, including our previous finding that people with and without obesity had similar hospitalization rates for CO-RSV.^15^ The lack of association of obesity with severe RSV contrasts with the association of obesity with severe influenza and COVID-19.^22^ Finally, in the current study, patients with HA-RSV were more likely have CKD compared to patients with CO-RSV. Similarly, other studies have reported that patients with HA-influenza had significantly higher rates of renal disease compared to those with CO influenza.^17,23^ In a post hoc analysis, we found that the LOS for patients with HA-RSV and CO-RSV who had CKD was significantly longer than those without CKD: median, 10 days (IQR, 6–25) versus 8 days (IQR, 3–14), respectively (*P* = .004). Patients with CKD may have had an increased opportunity for exposure to respiratory viruses while hospitalized.

Although most patients with HA-RSV did not have decompensation in their respiratory status, 29% of patients with HA-RSV had an escalation of respiratory support from day −2 to day +4 from RSV detection, 15% required transfer to the ICU, and 4% had initiation of mechanical ventilation temporally associated with RSV detection. Those who had an escalation of respiratory support were older and more likely to have respiratory comorbidities, further underscoring the impact of RSV on older adults and those with chronic respiratory conditions. Those who had an escalation were also more likely to have severe outcomes, including ICU admissions and in-hospital mortality, although we were unable to determine whether these outcomes were attributed to RSV or to other underlying medical conditions.

Our findings contribute to an increased understanding of the impact of RSV in hospitalized adults. A substantial proportion of the patients with HA-RSV were frail on admission, evidenced by the finding that 14% lived in skilled nursing facilities prior to hospitalization. Furthermore, patients with HA-RSV were hospitalized for a median of 12 days prior to detection of RSV, suggesting that their admitting diagnoses and comorbid conditions required prolonged hospitalizations. Although ICU admission and mechanical ventilation after RSV detection were similar among patients with HA-RSV and CO-RSV, patients with HA-RSV had overall longer lengths of hospital stay following RSV detection, had a higher proportion of deaths, and were frailer at discharge. A higher proportion of patients in the HA-RSV group required admission to a skilled nursing facility. A combination of underlying conditions, HA-RSV infection, and deconditioning or loss of functional status associated with prolonged hospitalization in older adults could have contributed to these findings,^24^ and they highlight the high level of healthcare utilization associated with HA-RSV and the need to prevent HA-RSV in hospitalized adults.

Our data suggest that using ICD-10 codes or death certificates would underestimate the burden of HA-RSV in adults because few of the patients with HA-RSV had RSV-related ICD-10 codes and none had RSV noted on their death certificate. This finding could reflect the relative lack of clinical impact of HA-RSV and/or the timing of death relative to detection of RSV infection, but it may also reflect an underappreciation of the potential impact of HA-RSV by providers and diagnostic coders. Similarly, in a study of adults admitted with respiratory illness and RSV detected by PCR, only 51% of patients had an ICD-10 discharge code corresponding to RSV infection.^25^ Others have suggested that the use of death certificates alone may significantly underestimate mortality associated with RSV infection.^26^

This study had several limitations. Given the relatively small sample size, the study may have been underpowered to detect differences in some outcomes between patients with HA-RSV versus CO-RSV. The study was also conducted in 2 academic centers; thus, these findings may not be generalizable to other settings. Although EMRs were queried for all RSV-positive patients during the study seasons, patients with HA-RSV were likely underestimated because testing for RSV in hospitalized patients with new onset respiratory symptoms was not systematic. Furthermore, patients were not tested and documented to be negative at admission; thus, CO-RSV may have been misclassified as HA because of prolonged detection from an illness prior to admission. Another potential limitation was that criteria for escalation of respiratory support were not standardized but were implemented at the discretion of providers. Finally, clinical outcomes cannot definitively be attributed to RSV versus other underlying medical conditions.

In conclusion, this study provides a unique perspective on the impact of HA-RSV in hospitalized adults. Healthcare-associated respiratory viruses are likely underappreciated in hospitalized adults. We found that HA-RSV was associated with escalation of respiratory support and an increased level of support in patients’ living situation at discharge. These outcomes increase the use of healthcare resources and related costs. Although this study cannot determine the mortality rate associated with HA-RSV, available data, such as death certificates and ICD-10 codes, likely underestimate this outcome. Infection control and prevention and RSV vaccines for adults at high risk of HA-RSV could mitigate this risk.
